# Repeated local delivery of hyaluronic acid gel as adjunctive treatment of residual pockets in periodontitis patients undergoing supportive periodontal care. A randomized controlled clinical trial

**DOI:** 10.1007/s00784-024-05505-9

**Published:** 2024-02-20

**Authors:** Kristina Bertl, Stefania Vlachou, Nikolaos Pandis, Antonios Zampelis, Andreas Stavropoulos

**Affiliations:** 1 Department of Periodontology, Dental Clinic, Faculty of Medicine, Sigmund Freud University Vienna, Freudplatz 3, 1020 Vienna, Austria; 2 Department of Periodontology, Blekinge Hospital, Hälsovägen, Byggnad 13, 371 41 Karlskrona, Sweden; 3 Division of Regenerative Dental Medicine and Periodontology, CUMD, University of Geneva, Rue Michel-Servet 1, 1211 Genève 4, Switzerland; 4 Department of Orthodontics and Dentofacial Orthopedics, School of Dental Medicine, University of Bern, Freiburgstrasse 7, 3010 Bern, Switzerland; 5 Private Practice, Drottninggatan 27, 652 25 Karlstad, Sweden; 6 Specialist Clinic for Endodontics and Periodontology, Public Dental Service, Värmland, Hagagatan 6, 652 20 Karlstad, Sweden; 7 Periodontology, Faculty of Odontology, University of Malmö, Carl Gustafs väg 34, 205 06 Malmö, Sweden; 8 Division of Conservative Dentistry and Periodontology, University Clinic of Dentistry, Medical University of Vienna, Sensengasse 2a, 1090 Vienna, Austria; 9 Department of Periodontology, School of Dental Medicine, University of Bern, Freiburgstrasse 7, Bern, 3010 Switzerland

**Keywords:** Adjunct treatment, Hyaluronic acid, Periodontitis, Pocket closure, Randomized controlled clinical trial, Supportive periodontal care

## Abstract

**Objectives:**

To assess the effect of hyaluronic acid (HyA) application as adjunct to re-instrumentation of residual pockets in patients undergoing regular supportive periodontal care (SPC).

**Methods:**

Chronic periodontitis patients (stage III and IV, grade B and C) with 4 interproximal residual pockets were randomly assigned to the test (HyA gel) or control (saline) group. After subgingival instrumentation, test or control substance was applied subgingivally, then daily supragingivally for 3 months, and if required a second time after subgingival re-instrumentation after 3 months. Clinical and patient reported outcome parameters were recorded every 3 months for 12 months. Pocket closure [probing pocket depth (PPD) ≤ 4mm with absence of bleeding on probing (BoP) at PPD = 4mm] was the main outcome parameter.

**Results:**

Fifty-six patients (221 experimental sites) were analysed. Pocket closure was achieved in 56.8 and 46.6% of the experimental sites in the test and control group, respectively (p > 0.05), while median PPD and PPD distribution (< 5mm/5mm/ > 5mm) differed significantly between groups in favour of the test group, at 12 months. Further, significantly fewer sites in the HyA group required re-instrumentation at 3 months, and sites in the HyA group showed a tendency for lower odds to remain diseased compared to the control group (OR 0.48, 95%CI 0.22–1.06). The odds for a site to remain diseased after 12 months increased significantly in the presence of plaque (OR 7.94, 95%CI 4.12–15.28), but in general, decreased significantly over time (OR 0.48, 95%CI 0.28–0.81).

**Conclusion:**

Re-instrumentation of residual pockets in SPC patients, per se*,* leads to a significant increase in pocket closure over time; this was impeded by poor plaque control. Repeated local application of HyA results in fewer sites requiring re-instrumentation and might slightly improve the rate of pocket closure. (clinicaltrials.gov registration nr. NCT04792541).

**Clinical relevance:**

HyA gel is easy to apply, well accepted by patients, and may have some positive effect in terms of fewer sites requiring re-instrumentation at 3 months and higher pocket closure rate at 12 months.

**Supplementary Information:**

The online version contains supplementary material available at 10.1007/s00784-024-05505-9.

## Introduction

Initial non-surgical subgingival instrumentation (i.e., second step of therapy), either by hand or (ultra)sonic instruments (or a combination thereof), is a very successful treatment approach in periodontitis patients; on average, a shallow probing pocket depth (PPD) can be achieved in 3 out of 4 pockets [1, 2]. Recently, the World Workshop on the Classification of Periodontal and Peri‐Implant Diseases and Conditions [3] and the EFP S3 level Clinical Practice Guideline [1] have defined the goal of periodontal treatment. On the site level, pocket closure is defined as PPD ≤ 4 mm and no bleeding on probing (BoP), while on the patient level, a successfully treated stable periodontitis patient is characterized by having 1) PPD of ≤ 4 mm, 2) no PPD = 4 mm with BoP, and 3) full-mouth BoP < 10%. Although this goal is difficult to achieve in every patient, it has been shown that patients reaching these clinical endpoints present a reduced rate of disease progression/recurrence and tooth loss due to periodontitis during long-term supportive periodontal care (SPC) [4, 5]. Hence, the use of adjunct products (locally or systemically delivered) during the second step of therapy is frequently discussed, with the aim to 1) further improve the rate of pocket closure, 2) reduce the need for additional surgical therapy, and/or 3) improve the rate of achieving a successfully treated stable periodontitis patient. This in turn, should not only improve long-term stability of treatment and reduce tooth loss, but also minimize patient morbidity and eventually treatment costs. The various options and the efficacy of locally delivered adjuncts (i.e., host modulators or antimicrobials) during the second step of therapy have been evaluated extensively within the frames of the EFP S3 level Clinical Practice Guideline [6, 7]. It was finally suggested that only locally applied sustained-released chlorhexidine and antibiotics may be considered as valid options [1, 6, 7].

In this context, there is limited information about the “best” timepoint of delivery of locally applied adjuncts, in terms of (cost-)efficiency, i.e., whether they should be applied during the second step of therapy or during the third or fourth step of therapy at re-instrumentation of residual/relapsing pockets, respectively. Considering patient, societal, and/or possible biological impacts of unnecessary use of any adjunct measure, the latter approach would allow to limit their use to those patients/sites, which do not heal after the initial subgingival instrumentation. Two recent systematic reviews covered the efficacy of adjuncts for periodontitis patients with residual/relapsing pockets during SPC [8, 9]. Specifically, among locally delivered antimicrobials, sustained-release chlorhexidine and tetracycline fibres, ranked highest with a statistically and clinically significant additional PPD reduction of approximately 0.6 to 0.7 mm [9], while insufficient evidence is available for any other adjunctive treatment option (i.e., other than antimicrobials) [8].

Another locally delivered adjunct, however, which has neither been included in any of the above-mentioned systematic reviews nor in the EFP S3 level Clinical Practice Guideline, is hyaluronic acid (HyA). Various gels containing HyA in different concentrations have been, since several years, tested as adjuncts to non-surgical mechanical subgingival instrumentation and their efficacy has even been summarized in few systematic reviews [10–12]. These reviews had well-comparable conclusions, i.e., most of the included clinical trials reported a positive, albeit moderate, effect in favour of HyA in terms of PPD and BoP reduction. However, there was a large variation in application modes and -frequency in the original studies, and all reviews stressed the need for further well-designed randomized controlled clinical trials (RCTs), including sufficient reporting of product details. In this context, it is currently not clear whether repeated application of a locally delivered adjunct is, in general, advantageous compared to single application, i.e., only just after instrumentation [13]. Nevertheless, 2 recent RCTs have assessed in-office HyA application specifically in re-instrumentation of residual/relapsing pockets. Each study tested a different HyA product and application frequency; in one of the studies HyA was applied subgingivally, once [14], while in the other study HyA was applied subgingivally at 2 consecutive appointments [15], but both showed a tendency in favour of HyA application.

Thus, it seems reasonable to assess whether combined local application of HyA, i.e., subgingivally in-office, after mechanical instrumentation, and repeated supragingivally, by the patient, once per day for 3 months, has the potential to improve the rate of pocket closure compared to re-instrumentation alone in the treatment of residual pockets in periodontitis patients undergoing regular SPC.

## Materials and methods

### Study design and participants

The present study was a randomized, double-blinded (patient and observer), placebo-controlled, parallel-group clinical trial with 12 months follow-up and is reported in accordance with the standards of reporting clinical trials (CONSORT Statement; Appendix 1) [16]. The protocol was approved by the regional ethical review board (Dnr. 2016/468) and registered at clinicaltrials.gov (NCT04792541); any changes in the protocol after its publication are listed in Appendix 2. Patient recruitment was performed between December 2016 and January 2020, i.e., patient recruitment started prior to the World Workshop on the Classification of Periodontal and Peri‐Implant Diseases and Conditions in 2017 [17, 18]. All periodontitis patients, that had completed the active treatment phase since ≥ 6 months and were enrolled in a regular SPC program (i.e., step 4 of treatment) in a Specialist Clinic for Periodontology (Public Dental Service, Värmland, Sweden), fulfilling the following inclusion criteria were consecutively included: (i) 35 to 75 years old, (ii) diagnosis of chronic periodontitis [19], (iii) at least 10 remaining teeth, and (iv) 4 to 8 interproximal sites with PPD of ≥ 5 to < 8 mm and presence of BoP at timepoint of inclusion. Further, the following exclusion criteria were defined: (i) molars with furcation involvement class II or III [20], (ii) class II or III tooth mobility, (iii) > 2 mm deeper PPD at another site in the same interproximal space (iv) antibiotic therapy in the preceding 6 months or during study participation, (v) need for antibiotic prophylaxis prior to periodontal examination and/or treatment, (vi) long-term use of anti-inflammatory and immunosuppressive medication, (vii) uncontrolled or newly diagnosed diabetes prior to or during study participation, (viii) pregnancy or lactation, (ix) severe occlusal dysfunction, (x) ongoing orthodontic treatment, and (xi) endodontic lesions. Age, gender, smoking status (i.e., never / former / current smoker) and presence/absence of a well-controlled diabetes mellitus was recorded for each participant.

### Test and placebo product

The product used in the “test/HyA group” for sub- and supragingival application was a gel containing hyaluronic acid (0.3%, non-crosslinked, middle molecular weight; Afta Clear™ Gel; Sunstar Europe SA, Etoy, Switzerland), while the “control/placebo group” received a small bottle of physiological saline solution. Both products were masked with white tape, so that the patient was unaware of group allocation.

### Intervention, randomization, and blinding

**Fig. 1:**
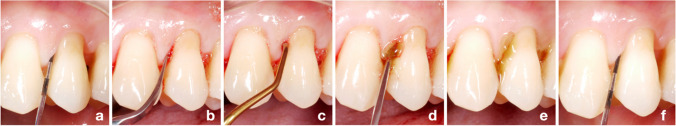
The experimental sites (**a**) received at the first supportive periodontal care session standard re-instrumentation with an ultrasonic device (**b**) and/or hand instruments (**c**) and after debridement the test or placebo product was applied subgingivally (in this specific case, the test product) (**d**–**e**); the patient returned for follow-ups after 3, 6, 9, and 12 months (**f**)

Prior to the beginning of the study a random sequence list for group allocation was computer generated (ratio 1:1 for the test/HyA and control/placebo group) by a periodontist not included in the recruitment process (KB). All participants were recruited by one of 3 experienced dental hygienists (LG, AS, MSS). Four interproximal sites of each participant were defined as experimental sites (i.e., mesio-buccal, mesio-palatal/-lingual, disto-buccal, or disto-palatal/-lingual); if possible 1 site per quadrant was chosen, otherwise the experimental sites had to be in different interproximal spaces. All participants received at baseline a standard SPC session including re-instrumentation with an ultrasonic device (E.M.S. Electro Medical Systems S.A., Nyon, Switzerland) and/or hand instruments (HuFriedyGroup, Chicago, USA) of all residual pockets by one of the above-mentioned 3 dental hygienists; each patient was treated by the same dental hygienist throughout the study period. Group allocation was concealed until after re-instrumentation of the residual pockets. After re-instrumentation, a periodontist (AZ) joined the treatment session, revealed group allocation only to himself by opening a hidden note in the envelope with the patient’s case report form, and applied either the test or placebo product subgingivally at all experimental sites. For subgingival application the test and placebo product were transferred into a sterile syringe with a blunt needle and the experimental sites were filled until the product was overflowing from the pocket (Fig. 1). The same periodontist performed this procedure to all participants, while the dental hygienists and patients remained blinded to group allocation. Each patient received a leaflet with a summary of the relevant instructions and a drawing indicating the 4 experimental sites including the correct size of interdental brushes (TePe Munhygienprodukter AB, Malmö, Sweden) for the specific interproximal space and either the test or placebo product. Patients had to apply the product once daily supragingivally with an interdental brush after tooth-brushing, only at the experimental sites, and for the coming 3 months until the second SPC appointment (i.e., 3-month follow-up). In addition, the patients were instructed to avoid tooth brushing or eating for 3 h after the application and not to use any mouth rinsing solutions or any other gels during participating in the present study. SPC sessions were repeated after 3, 6, 9, and 12 months, however, the patient stopped applying the test or placebo product after the first 3 months. Finally, all experimental sites with PPD = 5 mm and BoP or PPD > 5 mm at the 3-month follow-up received again re-instrumentation and a second subgingival application of the allocated product, as described above. In general, all sites with PPD = 5 mm and BoP or PPD > 5 mm at any of the SPC sessions received as a standard of care subgingival re-instrumentation.

### Outcome assessment and blinding

Subgingival microbiological sampling from the 4 experimental sites was performed at baseline and after 3, 6, and 12 months. The results of the microbiological sampling will be reported elsewhere. Further, after the microbiological sampling and at each SPC (i.e., at baseline, and after 3, 6, 9, and 12 months) the same blinded and calibrated dental hygienist (LG, AS, MSS) recorded PPD, clinical attachment level (CAL), presence/absence of BoP, and presence/absence of plaque at each experimental site. As a standard of care the periodontist (AZ) of this specific Specialist Clinic controls the PPD measurements of each hygienist approximately 2- to 3-times per year, which was considered as calibration. Further, at each follow-up patients were asked about any changes in their medical history, and about any intake of antibiotics in the preceding 3 months. At the second SPC (i.e., 3-month follow-up), the patients additionally received a short questionnaire about their opinion of the tested product. The questionnaire asked for the presence and intensity of pain during application, and for the patients’ opinion on the consistency and taste of the product during home use, using a scale from 1 to 10. A successful treatment outcome (i.e., pocket closure) was defined as PPD ≤ 4 mm with absence of BoP at PPD = 4 mm [1, 3]; if these criteria were not fulfilled the experimental site remained classified as “diseased”. This composite outcome was defined as primary outcome parameter and assessed at each SPC.

### Follow-up eligibility criteria

The patients were scheduled every 3 months for SPC, however, a range of 2.5 to 5 months between 2 SPC sessions was allowed; if the time between 2 SPC sessions exceeded 5 months, the SPC appointment was judged as missed. Further, the total period from the first to the fifth SPC appointment was not allowed to exceed 18 months, otherwise the patient was excluded. Due to having included several patients prior to the outbreak of the Covid-19 pandemic, patients were allowed to miss either the 6- or 9-month appointment without being excluded from the study. However, patients missing 2 SPC sessions or the 3- or 12-month appointment were also excluded.

### Sample size calculation

For the sample size calculation, it was assumed, that 75 and 25% of the sites in the test/HyA and control/placebo group, respectively, would achieve pocket closure, i.e., PPD ≤ 4 mm with absence of BoP at PPD = 4 mm [1, 3]. In the presence of these clinical parameters risk for disease progression is considered as low and treatment is considered as successful. Based on the data set of a previous publication [10] an intra-individual correlation coefficient of 0.42 is assumed for the presence of BoP. Based on this correlation coefficient, a power of 0.80, and an alpha value of 0.05, a sample size of 30 participants per group (i.e., total study population of 60) was calculated. To compensate for dropouts a total of 80 patients (40 patients per group) were consecutively recruited.

### Statistical analysis

Frequency distribution for categorical variables (such as gender, smoking status, PPD distribution, etc.) and means (standard deviations) or medians and interquartile ranges for continuous variables (such as age, PPD, etc.) are reported separately for the test/HyA and control/placebo group. To test for any differences between the test/HyA and control/placebo group either Fisher’s exact test or chi-squared test was applied for categorical parameters (i.e., chi-squared test was applied if each cell presented with a frequency > 5) and for continuous variables either an independent *t*-test (for normally distributed data) or a Mann Whitney-U test (for non-normally distributed data). Normality of the data was controlled by the Shapiro-Wilk test. The “health status of the experimental site” was defined as the primary outcome parameter [dichotomous; “diseased” (i.e., PPD > 4 mm or PPD = 4 mm with BoP) versus “successfully treated/pocket closure” (i.e., PPD ≤ 4 mm with absence of BoP at PPD = 4 mm). Two multivariable mixed-effects logistic regression models were calculated with the group allocation as the main predictor and the following a priori confounders: 1) tooth type (anterior / premolar / molar), 2) interproximal site (buccal / palatal/lingual), 3) PPD at baseline at the experimental site (5 mm / 6–7 mm), 4) plaque at the experimental site at each appointment (absent / present), 5) smoking status (never / former / current), 6) gender (female / male), 7) time passed between appointments (months), and 8) age (years). The first model included only the 3-month data, while the second model included all data collected over a 12-month period; in the latter model an additional confounder was added, i.e., timepoint (3- / 6- / 9- / 12-month follow-up). Statistical analysis was performed with STATA/IC 17.0 for Mac (Lakeway Drive, Texas, USA) and a p-value ≤ 0.05 was considered as statistically significant.

## Results

### Characteristics of the population at baseline (patient level)

**Fig. 2:**
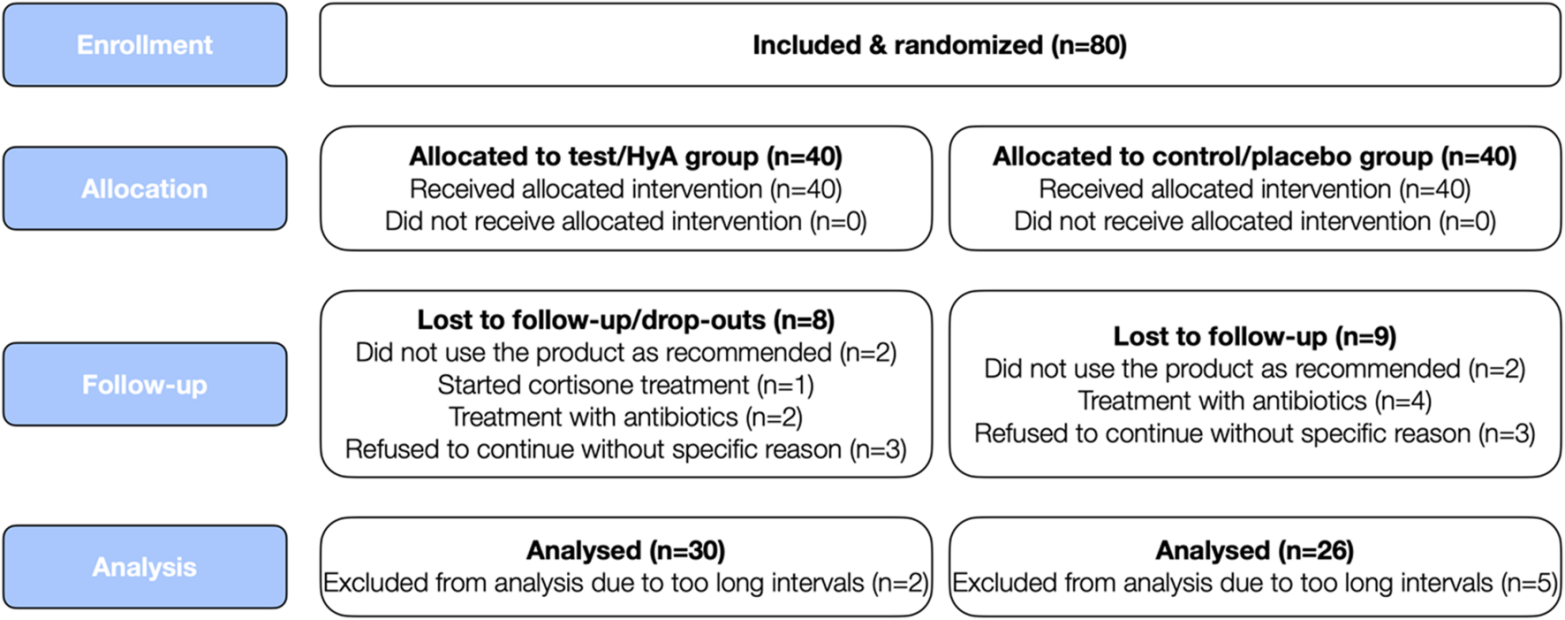
CONSORT flow diagram

Eighty chronic periodontitis patients (corresponding to periodontitis patients with stage III and IV and grade B and C) [18] undergoing regular SPC were recruited and randomized. Altogether, 24 participants dropped out or could not be included in the analysis due to various reasons (for details see Fig. 2). The characteristics of the 56 patients (i.e., 30 and 26 patients in the test/HyA and control/placebo group, respectively), contributing to the analysis, are displayed in Table 1; none of the baseline characteristics on the patient level differed significantly between the 2 groups. Shortly, the sample 1) consisted of ca. 66% females, 2) had an average age of 58 years, 3) included approximately 46% current smokers, and 4) had 3 well-controlled diabetics.

**Table 1:** Characteristics of the population and of the experimental sites at baseline (patient and tooth level)

Parameter		Test/HyA group (*n* = 30 patients with 118 sites)	Control/ placebo group (*n* = 26 patients with 103 sites)	*p*-value^1^
Patient level				
Gender [n (%)]	Female	20 (66.7)	17 (65.4)	0.920
Age	Mean (S.D.)	58.4 (8.7)	57.4 (8.5)	0.676
Smoking status [n (%)]	Never	7 (23.3)	11 (42.3)	0.347^2^
	Former	8 (26.7)	4 (15.4)	
	Current	15 (50.0)	11 (42.3)	
Diabetes [n (%)]	Present	2 (6.7)	1 (3.9)	1.000^2^
Tooth level				
Tooth type [n (%)]	Anterior	60 (50.9)	39 (37.9)	0.086
	Premolar	39 (33.0)	37 (35.9)	
	Molar	19 (16.1)	27 (26.2)	
Interproximal site [n (%)]	Buccal	57 (48.3)	59 (57.3)	0.183
	Palatal/lingual	61 (51.7)	44 (42.7)	
Plaque (at the experimental site) [n (%)]	Present	46 (39.0)	37 (35.9)	0.639
PPD (at the experimental site) [n (%)]	5 mm	101 (85.6)	92 (89.3)	0.406
	6–7 mm	17 (14.4)	11 (10.7)	
PPD (at the experimental site) (mm)	Median (Q1, Q3)	5 (5, 5)	5 (5, 5)	0.410^3^
	Mean (S.D.)	5.1 (0.4)	5.1 (0.4)	
CAL (at the experimental site) [n (%)]	5 mm	73 (61.9)	75 (72.8)	**0.042**^2^
	6–7 mm	42 (35.6)	22 (21.4)	
	8–9 mm	3 (2.5)	6 (5.8)	

Bold values indicate statistical significance

^1^ Statistical analysis does not take clustering of the data into account

^2^ Fisher’s exact test was applied

^3^ p-value relates to the median values and Mann Whitney-U test was applied

*CAL* clinical attachment level; *HyA* hyaluronic acid; *PPD* probing pocket depth; *Q1/3* first/third quartile, *S.D.* standard deviation

### Characteristics of the experimental sites at baseline (tooth level)

The sample included 221 experimental sites, which were all per eligibility criteria judged as “diseased” with presence of BoP at baseline. Except for 3 patients, each patient contributed with 4 experimental sites. Three experimental sites (one site each of 2 test/HyA patients and 1 control/placebo patient) had to be excluded due to rehabilitation with a new prosthetic restoration on the experimental or neighbouring tooth (*n* = 2) and due to one patient applying by mistake the product at the contralateral tooth, i.e., at the wrong one (*n* = 1). The baseline characteristics of the experimental sites of the test/HyA and control/placebo group are displayed in Table 1. Tooth type, interproximal sites, plaque, and PPD were well distributed among the 2 groups, while the test/HyA group contributed with significantly more sites displaying a CAL ≥ 6 mm compared to the control/placebo group (*p* = 0.042). Most of the sites presented with PPD = 5 mm at baseline, while 14.4 and 10.7% of the test/HyA and control/placebo group, respectively, had a PPD of 6 to 7 mm at baseline.

### Characteristics of the experimental sites after 3 months (tooth level)

The characteristics of the experimental sites after 3 months are reported in Table 2. After 3 months 44.1 and 34.0% of the experimental sites of the test/HyA and control/placebo group, respectively, achieved pocket closure without a significant difference between the groups. Similarly, the presence of plaque and BoP was comparable between the 2 groups. Yet, the median PPD (*p* = 0.003) and PPD distribution (< 5mm / 5 mm / > 5mm; *p* = 0.011) differed significantly between the groups in favour of the test/HyA group. Specifically, 61.9 and 41.7% of the experimental sites of the test/HyA and control/placebo group, respectively, presented with PPD < 5 mm. Furthermore, significantly less sites in the test/HyA group (*p* = 0.006) compared to the control/placebo group, 23.3 and 40.4%, respectively, required subgingival re-application at the 3-month follow-up.

**Table 2:** Characteristics of the experimental sites after 3 and 12 months (tooth level)

Parameter		Test/HyA Group (*n* = 118)	Control/ Placebo Group (*n* = 103)	*p*-value^1^
3-month follow-up				
Health status (at the experimental site) [n (%)]	Pocket closure	52 (44.1)	35 (34.0)	0.126
Requiring re-application [n (%)]	Yes	28 (23.3)	42 (40.4)	**0.006**
Plaque (at the experimental site) [n (%)]^3^	Present	22 (18.8)	29 (28.2)	0.101
BoP (at the experimental site) [n (%)]	Present	52 (44.1)	50 (48.5)	0.506
PPD (at the experimental site) [n (%)]	< 5 mm	73 (61.9)	43 (41.7)	**0.011**
	5 mm	39 (33.0)	53 (51.5)	
	> 5 mm	6 (5.1)	7 (6.8)	
PPD (at the experimental site) (mm)	Median (Q1, Q3)	4 (4, 5)	5 (4, 5)	**0.003**^2^
	Mean (S.D.)	4.4 (0.7)	4.6 (0.7)	
12-month follow-up				
Health status (at the experimental site) [n (%)]	Pocket closure	67 (56.8)	48 (46.6)	0.131
Plaque (at the experimental site) [n (%)]^4^	Present	20 (16.7)	12 (11.8)	0.277
BoP (at the experimental site) [n (%)]^4^	Present	39 (33.1)	30 (29.4)	0.562
PPD (at the experimental site) [n (%)]	< 5 mm	84 (71.2)	54 (52.4)	**0.007**
	5 mm	27 (22.9)	44 (42.7)	
	> 5 mm	7 (5.9)	5 (4.9)	
PPD (at the experimental site) (mm)	Median (Q1, Q3)	4 (4, 5)	4 (4, 5)	**0.007**^2^
	Mean (S.D.)	4.2 (0.9)	4.5 (0.8)	

Bold values indicate statistical significance

^1^ Statistical analysis does not take clustering of the data into account

^2^ p-value relates to the median values and Mann Whitney-U test was applied

^3^ One missing value in the test/HyA group

^4^ One missing value in the control/placebo group

*BoP* bleeding on probing; *HyA* hyaluronic acid; *PPD* probing pocket depth; *Q1/3* first/third quartile, *S.D.* standard deviation

### Assessment of the treatment outcome after 3 months

**Fig. 3:**
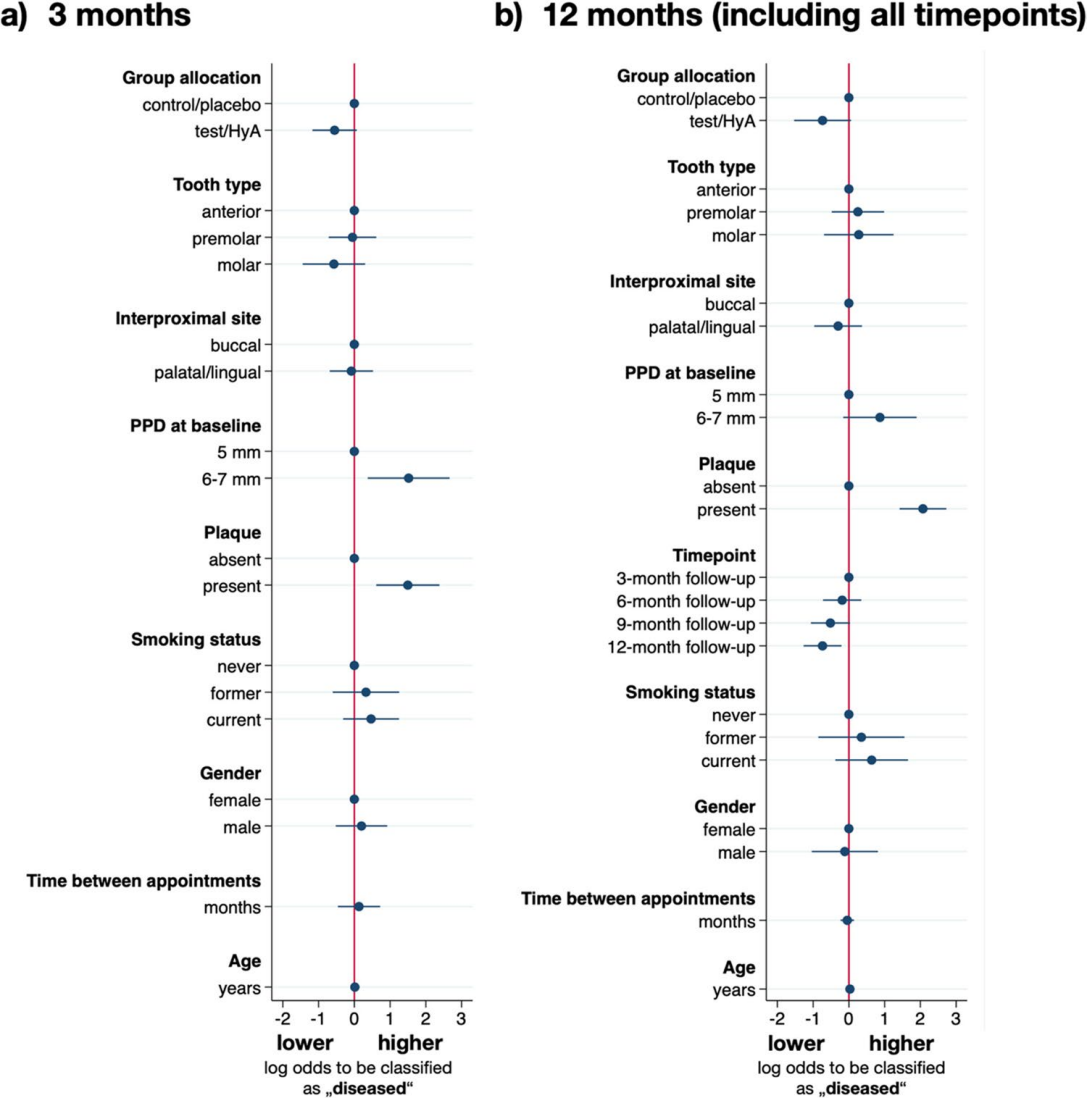
Results of the multivariable mixed-effects logistic regression analysis for the main dichotomous outcome parameter “health status of the experimental site” at a) the 3-month follow-up, and b) the 12-month follow-up (including all data from all timepoints). A log odds ratio above zero indicates higher odds for remaining diseased (i.e., PPD > 4 mm or PPD = 4 mm with BoP), while a log odds ratio below zero corresponds to higher odds of being successfully treated

The results of the multivariable mixed-effects logistic regression analysis after 3 months of SPC are displayed in Fig. 3a and Table 3. The experimental sites in the test/HyA group had slightly, but statistically non-significant lower odds compared to the control/placebo group to remain “diseased” (i.e., PPD > 4 mm or PPD = 4 mm with BoP) (OR 0.58, 95% CI 0.31–1.07; *p* = 0.081). Of the included confounders, only plaque and PPD at baseline had a significant effect on the outcome. Specifically, presence of plaque (OR 4.47, 95% CI 1.84–10.81; *p* = 0.001) and a higher PPD at baseline (OR 4.57, 95% CI 1.45–14.34; *p* = 0.009) significantly increased the odds to remain “diseased” at the 3-month follow-up.

**Table 3:** Results of the multivariable mixed-effects logistic regression analysis for the main dichotomous outcome parameter “health status of the experimental site” at the 3- and 12-month follow-up; an odds ratio above one indicates higher odds for remaining diseased (i.e., PPD > 4 mm or PPD = 4 mm with BoP)

Parameter		OR	95% CI		*p*-value
			Lower	Upper	
3-month follow-up					
Group allocation	Control/placebo	Ref			
	Test/HyA	0.576	0.309	1.071	0.081
Tooth type	Anterior	Ref			
	Premolar	0.949	0.488	1.846	0.877
	Molar	0.565	0.235	1.356	0.201
Interproximal site	Buccal	Ref			
	Palatal/lingual	0.921	0.502	1.688	0.789
PPD at baseline (at the experimental site)	5 mm	Ref			
	6–7 mm	**4.566**	**1.453**	**14.343**	**0.009**
Plaque (at the experimental site)	Absent	Ref			
	Present	**4.465**	**1.844**	**10.808**	**0.001**
Smoking status	Never	Ref			
	Former	1.384	0.546	3.510	0.494
	Current	1.597	0.730	3.491	0.241
Gender	Female	Ref			
	Male	1.221	0.595	2.506	0.586
Time between appointments	Months	1.139	0.631	2.056	0.666
Age	Years	1.015	0.978	1.053	0.432
12-month follow-up					
Group allocation	Control/placebo	Ref			
	Test/HyA	0.479	0.216	1.062	0.070
Tooth type	Anterior	Ref			
	Premolar	1.287	0.618	2.679	0.501
	Molar	1.319	0.499	3.489	0.577
Interproximal site	Buccal	Ref			
	Palatal/lingual	0.740	0.380	1.444	0.378
PPD at baseline (at the experimental site)	5 mm	Ref			
	6–7 mm	2.385	0.854	6.658	0.097
Plaque (at the experimental site)	Absent	Ref			
	Present	**7.938**	**4.122**	**15.284**	** < 0.001**
Timepoint	3-month follow-up	Ref			
	6-month follow-up	0.830	0.485	1.418	0.494
	9-month follow-up	0.597	0.345	1.033	0.065
	12-month follow-up	**0.479**	**0.282**	**0.813**	**0.006**
Smoking status	Never	Ref			
	Former	1.419	0.425	4.732	0.569
	Current	1.892	0.685	5.230	0.219
Gender	Female	Ref			
	Male	0.893	0.354	2.254	0.810
Time between appointments	Months	0.957	0.792	1.156	0.646
Age	Years	1.031	0.984	1.081	0.196

Bold values indicate statistical significance

*BoP* bleeding on probing; *CI* confidence interval; *HyA* hyaluronic acid; *OR* odds ratio; *PPD* probing pocket depth

### Characteristics of the experimental sites after 12 months (tooth level)

The characteristics of the experimental sites at final evaluation (12-month follow-up) are reported in Table 2. At final evaluation 56.8 and 46.6% of the experimental sites of the test/HyA and control/placebo group, respectively, achieved pocket closure without a significant difference between the groups. Similar, the presence of plaque and BoP were comparable between the 2 groups. Yet, the median PPD (*p* = 0.007) and PPD distribution (< 5mm / 5 mm / > 5mm; *p* = 0.007) differed significantly between the groups in favour of the test/HyA group. Specifically, 71.2 and 52.4% of the test/HyA and control/placebo sites, respectively, presented with PPD < 5 mm and the median PPD was lower in the test/HyA group. In the test/HyA group, out of 28 experimental sites receiving re-application at 3 months, 28.6% were not diseased anymore after 12 months, while out of 90 experimental sites not receiving re-application at 3 months, 34.4% were classified as “diseased” again. In the control/placebo group, out of 42 experimental sites receiving re-application at 3 months, 33.3% were not diseased anymore after 12 months, while out of 61 experimental sites not receiving re-application at 3 months, 44.3% were classified as “diseased” again.

### Assessment of the treatment outcome after 12 months

The results of the multivariable mixed-effects logistic regression analysis are displayed in Fig. 3b and Table 3. The experimental sites of the test/HyA group had slightly, but statistically non-significant lower odds compared to the control/placebo group to remain “diseased” (i.e., PPD > 4 mm or PPD = 4 mm with BoP) (OR 0.48, 95% CI 0.22–1.06; *p* = 0.070). Of the included confounders, only plaque and timepoint had a significant effect on the outcome. Specifically, the presence of plaque significantly increased the odds to remain “diseased” by approximately 8-times (OR 7.94, 95% CI 4.12–15.28; *p* < 0.001), and, in general, the odds to remain “diseased” decreased during the study period reaching significance at the final evaluation (OR 0.48, 95% CI 0.28–0.81; *p* = 0.006).

### Patients’ opinion

**Fig. 4:**
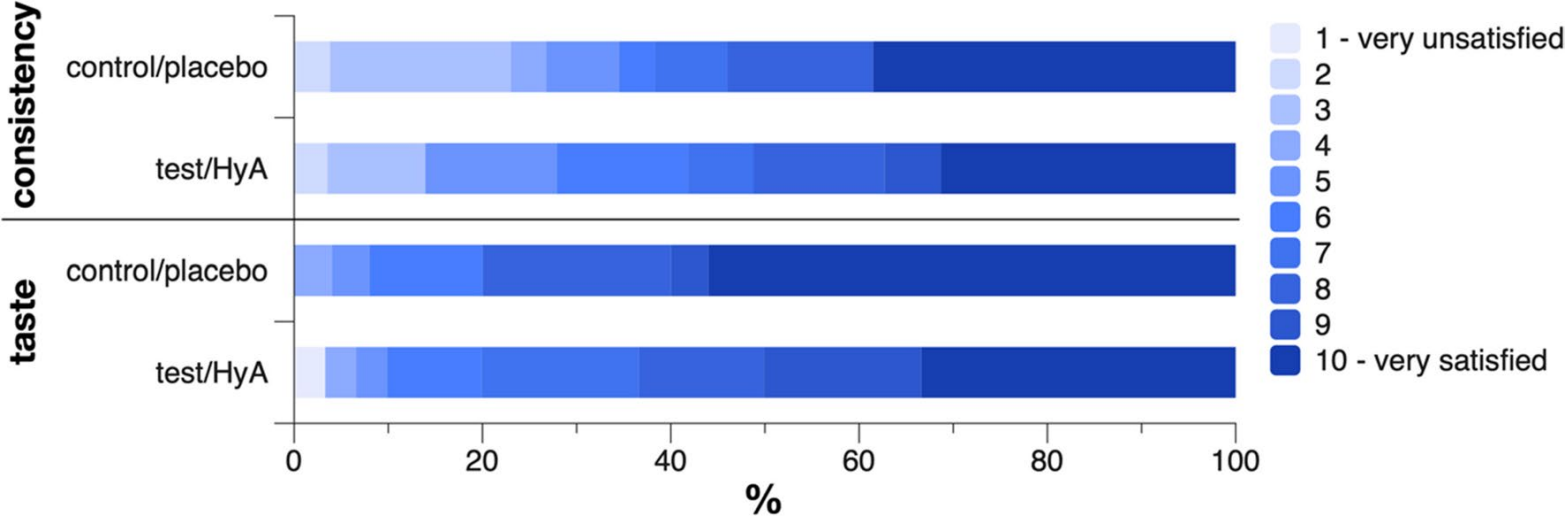
Patients’ opinion on the consistency and taste of the product evaluated on a scale from 1 (very unsatisfied) to 10 (very satisfied). Data represent the percentage of the patients judging each level of the scale

One patient of each group reported to have experienced pain at the gingiva during product application with an intensity of 3 to 4 out of 10, with 10 representing maximum pain. One patient of the test/HyA group did not answer on the consistency and one patient of the control/placebo group did not answer on the taste. The groups presented no significant difference in their opinion on the consistency and taste of the product (Fig. 4). Specifically, the mean values (standard deviation) of the scale for the consistency were 7.2 (2.6) and 7.0 (3.0) in the test/HyA and control/placebo group, respectively (*p* = 0.822), and the median values (first, third quartile) of the scale for the taste were 8.5 (7, 10) and 10 (8, 10) in the test/HyA and control/placebo group, respectively (*p* = 0.184); for both parameters a value of 10 represented being very satisfied.

### Safety

None of the patients reported any side effects/complications related to product application and no adverse events could be observed clinically.

## Discussion

Locally delivered chemotherapeutics are frequently used as adjunct to non-surgical treatment in periodontitis patients, either at the initial phase of treatment or at residual/relapsing pockets during a later stage of treatment. However, as initial non-surgical subgingival instrumentation is a rather successful treatment per se, a more site-specific approach for pockets that did not heal during the second step of therapy, and/or of relapsing pockets during SPC might be the more relevant approach. Herein, a HyA containing gel, repeatedly applied (daily) for a period of 3 months, as adjunct to re-instrumentation of residual/relapsing pockets in patients undergoing regular SPC, showed some tendency to improve the outcome, i.e., 10% higher rate of pocket closure was observed for the sites receiving HyA compared to those in the control group that received physiological saline solution (i.e., 57 versus 47% of the sites, respectively), after 12 months. Additionally, the percentage of experimental sites with PPD < 5 mm was about 1.4-times higher in the test/HyA group compared to the control/placebo group (i.e., 71 and 52% of the sites, respectively).

These results are in agreement with those reported in 2 recent RCTs [14, 15] assessing a HyA containing gel as adjunct to re-instrumentation of residual/relapsing pockets and showing that the use of a HyA containing gel resulted in a tendency for superior results compared to re-instrumentation only. Moreover, both studies reported in their test group a similar frequency of sites with PPD < 5 mm after 12 months as herein, i.e., 76 to 77% in the 2 previous studies and 71% herein [14, 15]. Further, in one of the RCT studies [15] reporting on pocket closure (i.e., PPD ≤ 4 mm without BoP), a comparable rate in the HyA group as herein was observed, i.e., in 59 and 57%, respectively. A significant difference, however, between those 2 RCTs and the current study regard the product characteristics of the applied HyA gel. Specifically, a non-crosslinked, middle molecular weight HyA with a relatively low concentration (0.3%) was used in this study, while the other studies either used a product with crosslinked, high-molecular HyA at a higher concentration [15] or a combination product with polynucleotides and high-molecular HyA at a higher concentration [14]. Laboratory studies on periodontal cells or periodontitis-associated pathogens have compared the effects of either non-crosslinked and crosslinked HyA [21–23] or of HyA of different molecular weights [24]; none of the formulations tested showed any negative effect in terms of periodontal wound healing. In this context, due to the lack of comparative clinical trials up-to-now, it remains unclear whether different HyA characteristics indeed translate into clinically relevant differences. Another difference among those 2 RCTs and herein regard the application mode/frequency of HyA. In this study, the HyA containing gel was repeatedly (daily) applied supragingivally for 3 months by the patients in addition to the subgingival in-office applications. One of the previous studies [15] assessed the effect of a repeated in-office application after 3 months but failed to show any additional beneficial effect of HyA after the re-application; however, prior to re-application no additional subgingival instrumentation was performed in this specific study. Herein, only sites with remaining signs of pathology received a re-application in combination with subgingival re-instrumentation. In both groups (i.e., test and control) approximately 30% of the sites requiring re-instrumentation and re-application were judged as successfully treated at the end of the trial. Nevertheless, although re-treatment improved the situation in 1 out of 3 cases and no distinct beneficial effect of HyA could be noted, the number of sites requiring re-instrumentation (re-treatment) at 3 months, was significantly lower in the HyA group, comparing with the one receiving NaCl. Finally, also the current data do not allow any conclusion on any potential additional effect of repeated supragingival application by the patients for 3 months, as a third group with subgingival in-office delivery only was lacking herein.

In general, reducing the number of residual pockets after active periodontal treatment (i.e., step 1 to 3) has been shown beneficial in terms of disease recurrence/progression and tooth loss in various studies focusing on long-term SPC after active periodontal treatment [4, 25–29]. Thus, it is reasonable to assume that any treatment measure, such as the use of locally applied adjuncts, that improve the rate of pocket closure – irrespective the stage of treatment – may contribute positively also to the long-term outcome. In this context, HyA containing gels as adjunct to subgingival instrumentation in the second step of therapy have shown some potential in terms of PPD and BoP reduction [10–12], which has been re-confirmed by more recent RCTs [30–33].

Compared to other studies with a similar study design but assessing different products as adjuncts to re-instrumentation of residual pockets in SPC patients, the frequency of pocket closure appeared a bit lower herein. For example, 2 recent RCTs assessing the adjunctive effect of flapless application of enamel matrix derivatives [34], sodium hypochlorite gel [35], and chlorhexidine gel [35] reported pocket closure (i.e., PPD ≤ 4 mm without BoP) in 80, 78, and 63% of the sites compared to 57% herein. Hence, and considering the lack of RCTs directly comparing these products, HyA appeared similarly effective to CHX gel, but slightly inferior to enamel matrix derivatives and sodium hypochlorite gel. The latter might be of specific interest, as the combination of sodium hypochlorite gel with crosslinked HyA gel has recently received attention as combined adjunctive treatment in periodontitis patients. However, a recent retrospective case series including 29 SPC patients with residual/relapsing pockets reported for this combined approach a somewhat lower pocket closure rate of 25% [36].

Among the confounders used for adjusting of the present analysis, two had a significant effect on the outcome at final evaluation, i.e., plaque control and compliance/time, which are both well in agreement with the literature. Specifically, herein the presence of plaque, which was assessed at each experimental site at each SPC session, significantly lowered the odds of achieving pocket closure. To successfully motivate patients for continuous high levels of plaque control during SPC is a well-known clinical challenge and recent long-term SPC studies re-confirmed the clinical relevance of it [4, 37]. Both studies, including 100 [4] and > 200 individuals [37], respectively, undergoing approximately 10 years of SPC after active periodontal treatment, showed an increased risk for disease recurrence and tooth loss due to periodontitis with poor plaque control. Further, the patients of the present RCT showed in general and independent of group allocation a significant improvement over time, which became significant after 12 months. Specifically, also in the control/placebo group almost 50% of the experimental sites achieved pocket closure at the end of the trial. This effect size of achieving pocket closure by mechanical re-instrumentation only is well comparable with previous studies reporting rates of 42 to 60% [34, 35, 38]. In addition, study participation per se probably has a beneficial effect on the compliance from a patient perspective but also from the perspective of the treating dentist/dental hygienist, who might be more insisting on keeping the 3-month interval. In this context, it has been shown, that a shorter time between consecutive SPC sessions can result over time in reduced PPD and periodontal stability, while a longer time may lead to periodontal instability and subsequently tooth loss [39].

The present study – performed under “real-life” conditions – comes with some limitations, such as a relatively high drop-out rate of 30%, which did not permit us reaching the intended sample size in the control/placebo group, i.e., 26 instead of 30 participants were analyzed. The high drop-out rate is at least partly owed to participants missing their scheduled appointments during the Covid-19 pandemic, i.e., although a range of 2.5 to 5 months between 2 SPC sessions was accepted, almost 10% of the participants (i.e., 2 and 5 of the test/HyA and control/placebo group, respectively) were excluded from the analysis due to too long intervals. Nevertheless, as the time between 2 SPC sessions appears as a relevant factor [39], this parameter was included in the regression analyses herein, to correct in the model the slight variation in SPC intervals. However, as the present trial was performed in parallel-group design, the patients did not receive both products, which in turn should limit any bias due to insufficient patient blinding. In this context it was interesting, that the groups presented no significant difference regarding patient opinion about the consistency and taste of the product; both products were well accepted by the patients, which in turn may indicate good compliance.

## Conclusion

Re-instrumentation of residual pockets in SPC patients, per se*,* leads to a significant increase in pocket closure over time. This improvement was dependent of the patient’s oral hygiene, i.e., the presence of plaque increased the odds to remain diseased by approximately 8-times. The additional sub- and supragingival repeated application of a HyA containing gel resulted in significantly fewer sites requiring re-instrumentation at 3 months, and into some clinically relevant differences after 12 months of SPC compared to the control/placebo group, such as 71 versus 52% of experimental sites reaching a PPD < 5 mm, respectively. However, statistical significance of this effect was marginally missed in the adjusted analysis on achieving pocket closure. Hence, further clinical trials are needed to confirm superiority of this adjunct compared to subgingival re-instrumentation alone.

### Supplementary Information

Below is the link to the electronic supplementary material.

Supplementary file1 (DOCX 875 KB)

## Data Availability

No datasets were generated or analysed during the current study.
