# Supplementary material for: Repeated local delivery of hyaluronic acid gel as adjunctive treatment of residual pockets in periodontitis patients undergoing supportive periodontal care. A randomized controlled clinical trial
Source: Clin Oral Investig. 2024 Feb 20;28(2):158. doi: 10.1007/s00784-024-05505-9 (PMC10879318; doi:10.1007/s00784-024-05505-9)
Supplement: Supplementary file 1 — Supplementary file1 (DOCX 875 KB) [file 784_2024_5505_MOESM1_ESM.docx]

**Appendix legends**

**Appendix 1.** CONSORT checklist.

**Appendix 2.** Changes in the protocol after its publication on clinicaltrials.gov.

The following aspects have been modified after publication of the protocol on clinicaltrials.gov:

1. The sample size was increased to 80 (instead of 66) patients to ensure the intended sample size despite drop-outs occurring over a 12-month period.
2. The outcome parameter was adjusted after the World Workshop on the Classification of Periodontal and Peri‐Implant Diseases and Conditions in 2017. The original definition of pocket closure was probing pocket depth (PPD) ≤ 5 mm with absence of bleeding on probing (BoP) at PPD = 5 mm, which was adapted to the new criteria of “successfully treated”, which are “PPD ≤ 4 mm with absence of BoP at PPD = 4 mm”. However, this did not affect our sample size calculation, which is based on a percentage of “successfully treated” experimental sites (i.e., sites with pocket closure) per patient independent of the exact definition.
